# Ultrafast electron crystallography of the cooperative reaction path in vanadium dioxide

**DOI:** 10.1063/1.4953370

**Published:** 2016-06-06

**Authors:** Ding-Shyue Yang, Peter Baum, Ahmed H. Zewail

**Affiliations:** 1 Department of Chemistry, University of Houston, Houston, Texas 77204, USA; 2 Ludwig-Maximilians-Universität München, Am Coulombwall 1, 85748 Garching, Germany; 3 Physical Biology Center for Ultrafast Science and Technology, Arthur Amos Noyes Laboratory of Chemical Physics, California Institute of Technology, Pasadena, California 91125, USA

## Abstract

Time-resolved electron diffraction with atomic-scale spatial and temporal resolution was used to unravel the transformation pathway in the photoinduced structural phase transition of vanadium dioxide. Results from bulk crystals and single-crystalline thin-films reveal a common, stepwise mechanism: First, there is a femtosecond V−V bond dilation within 300 fs, second, an intracell adjustment in picoseconds and, third, a nanoscale shear motion within tens of picoseconds. Experiments at different ambient temperatures and pump laser fluences reveal a temperature-dependent excitation threshold required to trigger the transitional reaction path of the atomic motions.

## I. INTRODUCTION

Vanadium dioxide is a strongly correlated material with a first-order thermodynamic transition at around 340 K between an insulating phase of monoclinic crystallographic symmetry and a metallic phase with a tetragonal (rutile) crystal structure.^1–3^ Dynamically, via photoexcitation above the bandgap,^4^ the structural phase transition has been resolved on the atomic spatiotemporal scale to proceed along a stepwise reaction path, where the atoms move from the initial over transitional to final conformations with a hierarchy of time scales ranging from femtoseconds (fs) to hundreds of picoseconds (ps).^5^ Different aspects of this path require different degrees of cooperation, evident from the distinct energy thresholds below which the structural phase transition does not fully proceed.^5,6^ Spectroscopic methods using lasers or terahertz radiation have also revealed such a threshold for the electronic and phononic aspects of the transition, and related time scales.^7–12^ Overall, there is a cooperative, sequential reaction path in which the structural and electronic degrees of freedom are intimately related.^13^

Here, we report a femtosecond electron diffraction study of the structural dynamics in VO_2_, covering a larger scope of excitation parameters, sample morphologies, and probe geometries than in our initial report.^5^ We especially concentrate our analyses on the temperature dependence of the fluence threshold, in order to examine the relation between the thermodynamic aspects and the nonequilibrium pathway of the phase transition. First, we find that the stepwise structural reaction, initially discovered in a macroscopic single crystal^5^ and later in a free-standing membrane,^6^ is also present in ∼100-nm-thick films deposited on a substrate. The measured times, atomic motion directions, and fluence thresholds are similar in all cases, indicating the general nature of the ultrafast reaction mechanism. On the ps time scale and longer, however, where equilibration over all degrees of freedom prevails, there must be an influence of the ambient temperature before excitation. We therefore compare here excitation threshold measurements at different ambient temperatures. The results indicate, for both the bulk and the thin-film crystals, that a temperature-dependent laser fluence threshold is required to properly initiate the structural pathway. This observation for the structural transformation matches earlier findings on the metal-insulator transition by spectroscopy^7,10^ and supports the initial conjecture^4,5^ that the laser-induced and thermal pathways are intimately related.

## II. VO_2_ CRYSTALLOGRAPHY AND BASIC STRUCTURAL DYNAMICS

**FIG. 1.**
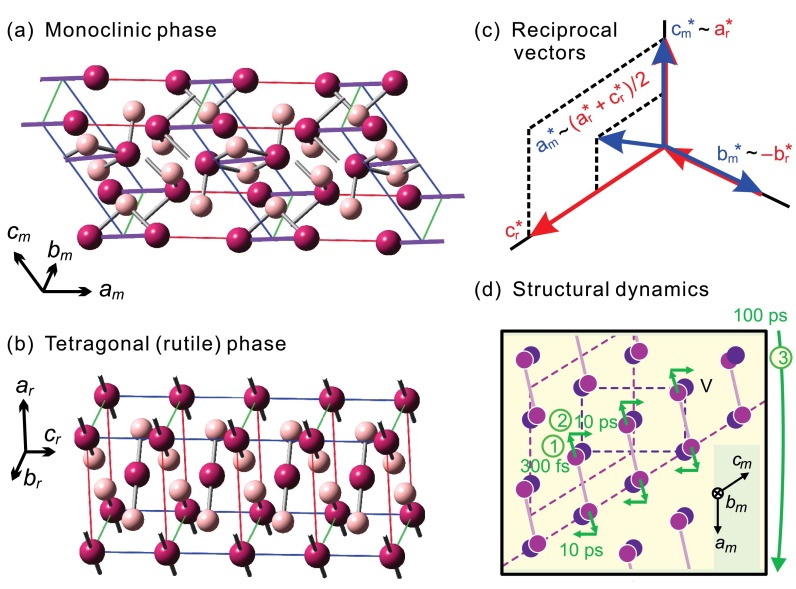
Crystal structures and time-resolved dynamics of VO_2_. (a) Structure of the monoclinic, low-temperature phase, where vanadium atoms are depicted in purple and oxygen atoms in pink. The V–V dimers are highlighted by violet lines. (b) Structure of the tetragonal (rutile), high-temperature phase. The distances between adjacent vanadium atoms are equal. (c) The relationship between the reciprocal basis vectors for the two phases (m: monoclinic; r: rutile). (d) The stepwise mechanism for the photoinduced structural dynamics, where the steps for the initial V–V bond breaking in 300 fs, the intracell reorganization in 10 ps, and the shear motion toward the final structure in 100 ps are indicated.

The two thermodynamically stable structures involved in the phased transition of VO_2_ are depicted in Fig. 1. Below the transition temperature, which is about 340 K for stoichiometric single crystals, the vanadium atoms arrange into pairs in a monoclinic phase *M*_1_ (Fig. 1(a)),^14^ whereas above the transition temperature all adjacent V–V distances are equal and the symmetry becomes tetragonal/rutile (Fig. 1(b)).^15^ By convention, the crystal axes are assigned differently and the *a* axis of the *M*_1_ phase matches with the *c* axis of the tetragonal phase; that is in real space, $a_{m}∼2c_{r}$, $b_{m}∼-b_{r}$, and $c_{m}∼a_{r}-c_{r}$, where the subscripts m and r refer to the *M*_1_ and rutile structures, respectively. Figure 1(c) shows the basis vectors in reciprocal space. Any reciprocal lattice point (*h*,*k*,*l*)_m_ of the *M*_1_ phase corresponds to $\left(\frac{h}{2}+l,\bar{k},\frac{h}{2}\right)$ of the tetragonal phase, except for those with an odd *h*. Thus, in diffraction, Bragg spots from the *M*_1_ phase with an odd *h* index become forbidden after the structural transformation; all other diffractions are in principle allowed, albeit with certain changes in their intensities and slight shifts in their positions due to intracell rearrangements and slight modifications of the lattice.^16^

Laser excitation at ∼1.5 eV with a sufficient energy density can trigger a sequence of events that ultimately, through distinct time scales and transitional structures, transform the material to the high-temperature phase (see Fig. 1(d)).^5,6^ First, after the electronic excitation, the vanadium dimers relax or delocalize within ∼300 fs. Second, the unit cell approaches the tetragonal-like atomic positions within several ps. Third, it takes tens to hundreds of ps to overcome the latent heat and arrive at the thermodynamically stable high-temperature phase. Since the metallic-like behavior may be reached within tens of fs,^13,17,18^ there is at least one type of metastable structure on the reaction path, which has been termed a transient metallic monoclinic state and has been subject of intensive studies.^7–10,18–21^

While investigating macroscopic, stoichiometric single crystals produces the cleanest picture of fundamental physics, most technological applications of VO_2_'s insulator-to-metal transitions, for example, as switches or memory, are more readily realized with thin-films that are coated on a substrate. A topic of this report is therefore whether the structural reaction path is different in such case, where one nanoscale dimension is involved, and whether fluence thresholds and their temperature dependence are the same. Since dynamics in VO_2_ is notoriously affected by stoichiometric deviation, defects, sample morphology, domain sizes and many other effects, we investigated in this work exclusively single-crystalline materials, i.e., bulk crystals or epitaxial thin-films.

## III. METHOD: ULTRAFAST ELECTRON CRYSTALLOGRAPHY (UEC)

**FIG. 2.**
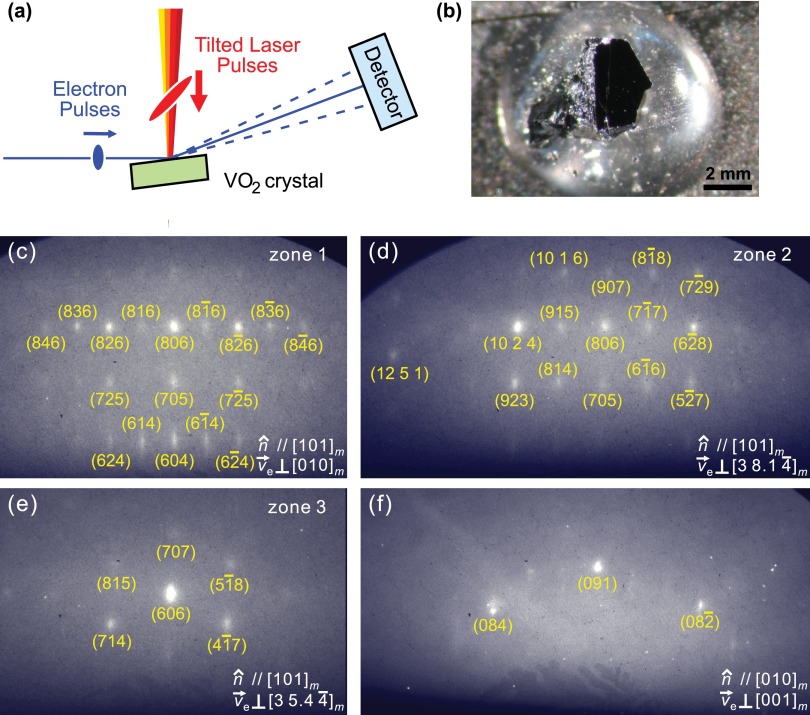
Experimental setup and diffraction patterns of VO_2_ single crystals observed by UEC. (a) Tilting of the excitation laser pulses to match with the footprint of the electron probe pulses for a femtosecond temporal resolution. The electron incidence angle was ∼5°. (b) Single crystal of VO_2_ with a polished (403)_m_ surface. (c)–(f) Diffraction patterns observed from two different crystal surfaces and different zone axes, where $\hat{n}$ is the surface normal direction and ${\vec{v}}_{e}$ is the electron propagation direction (zone axis).

Tracking atomic motion on the temporal and spatial scales relevant to photoinduced phase transitions requires femtosecond and picometer resolutions, respectively. We achieved this using ultrafast electron crystallography (UEC).^22^ Figure 2(a) depicts how this experiment works in the case of bulk materials or surfaces. Ultrashort electron pulses at an energy of 30 keV, generated from a laser-driven photocathode, were collimated using a magnetic lens and directed onto the sample at a grazing incidence angle, here about 2°–5°. Laser excitation was made with tilted optical pulses to avoid velocity mismatch and propagation effects.^23,24^ Electron diffraction patterns were recorded at different pump-probe delay times on a phosphor screen coupled to an intensified CCD camera. In the fluence-dependent measurements, the laser pulse energy was adjusted by rotating a half-wave plate against a fixed polarizer to maintain the same optical polarization within the tilting optics and at the sample.

For the detection of primary femtosecond dynamics, we reduced the number of electrons per pulse to ∼500, below the space-charge limit^25^ and close to the duration limit^26^ of single-electron pulses^27,28^ without compression. At such a low flux, the electron pulse width has been measured as 322 ± 128 fs,^29^ which is only a little longer than achievable with work-function matching.^30^ For measuring slower dynamics, the use of packets with up to 3000 electrons per pulse was found more convenient. The temporal coincidence of the tilted laser excitation with the electron pulses (time zero) was determined by the multi-photon emission of a space-charge cloud from a needle tip, which is accurate to about ±1 ps.^23^

## IV. RESULTS AND DISCUSSION

### A. VO_2_ bulk single crystals

Figure 2(b) shows a picture of the blackish VO_2_ single crystal used in the first part of the reported experiments. Bulk crystals with a size of about 2 × 2 × 2 mm^3^ were grown by the vapor transport method^31^ and were from the same source as those samples in Refs. 32 and 33. Static X-ray diffraction with graphite-monochromated Mo K_α_ radiation (Bruker SMART 1000 diffractometer) confirmed the expected crystal structures below and above the phase-transition temperature of 340 K and revealed a long-range order and stoichiometry better than measurable.

Figures 2(c)–2(e) show the electron diffraction patterns obtained at different azimuthal orientations of the crystal surface, which was mechanically cut, polished, and rinsed with acetone. Alternatively, a few of the naturally grown crystal surfaces readily produced a pattern (Fig. 2(f)). While time-resolved reflection high-energy electron diffraction can well reveal surface-dominated dynamics,^34^ we here study largely the bulk material, by measuring rough surfaces and Bragg reflections with rather high Miller indices at incidence angles of ∼5°. This is evident from the transmission-like diffraction patterns (Fig. 2) with multiple and narrow spots. Electrons at 30 keV have a mean-free-path length of ∼15 nm in VO_2_ and an inelastic mean free path of ∼27 nm. The transmission-like diffraction pattern can therefore be understood as a combination of the large-angle incidence and surface roughness from the mechanical polishing procedure, which apparently produces single-crystalline surface islands of tens-of-nanometer size. The thermally induced phase transition was directly observed in the electron diffraction experiment when the material was heated, revealing for the intensity of rutile-forbidden Bragg spots at 340 K a hysteresis width of <6 K, the upper limit due to the applied heating and cooling rates (∼0.6 K/min). The typical hysteresis of our single crystals is much smaller; see the inset of Fig. 2 in Ref. 33.

In the pump-probe experiments, the laser excitation spot (800-nm wavelength, 1.55-eV photon energy, 1-kHz repetition rate, 120-fs pulse duration) was made about twice the size of the area probed by the electron beam, which was 2 mm (the sample size) by 0.2 mm (the electron beam diameter). Within the experimental repetition period of 1 ms at 1 kHz, we observed a full back reaction and recovery to the initial *M*_1_ phase in all types of VO_2_ samples, which was concluded from observing no change between the diffraction patterns recorded at negative delay times (effective a 1-ms probe time) and those recorded without any excitation. Thus, contributions from static laser heating can be excluded, and the observed diffraction changes exclusively reveal the nonequilibrium dynamics following the femtosecond excitation. We also confirmed the absence of photoinduced transient electric fields and charging from the observation of a steady position and intensity of the direct, non-diffracted electron beam for all delay times.

**FIG. 3.**
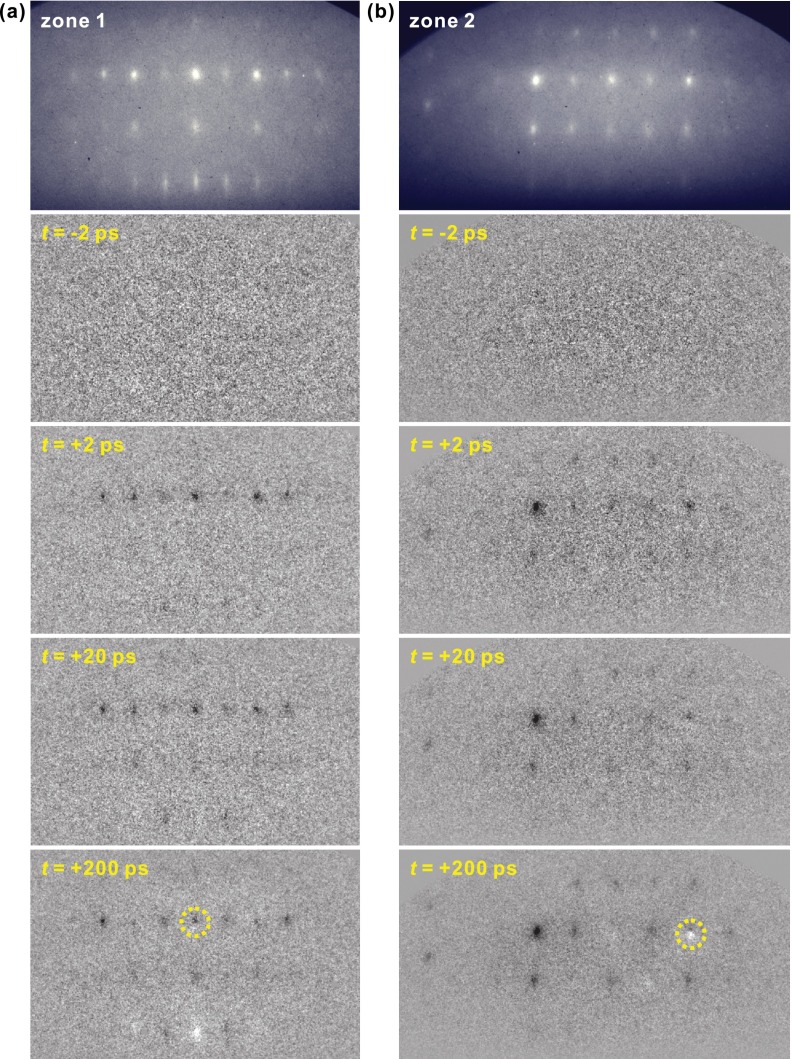
Diffraction patterns of VO_2_ bulk at two zone axes (see Fig. 2, panels (c) and (d)) and their time-dependent changes referenced to negative time frames, following the femtosecond optical excitation. No diffraction difference is observed before the zero of time. At positive times, dark spots signify intensity depletion and white spots intensity increase; the observation of both dark and white parts for the circled spots at *t* = 200 ps signifies a small position shift of the Bragg diffractions.

Figure 3 shows for two zone axes in the upper panels the basic diffraction, and below the differences to that pattern at selected probe delay times. There are tens of well-indexed Bragg spots, and 30 of them were clear enough for time-resolved investigations. After only ∼2 ps, a significant decrease in intensity with no distinct spot movement is observed for some Bragg diffractions; this intensity drop remains at a similar level for ∼20 ps. Within 200 ps, further diffraction changes develop, such as continuing intensity decreases or increases or spot position shifts (e.g., circled differences in Fig. 3). The resulting diffraction patterns then remain steady for the following 1 ns, the longest time recorded, and return to the original pattern within <1 ms. These distinct time scales signify the sequential nature of the photoinduced structural transformation of VO_2_.

**FIG. 4.**
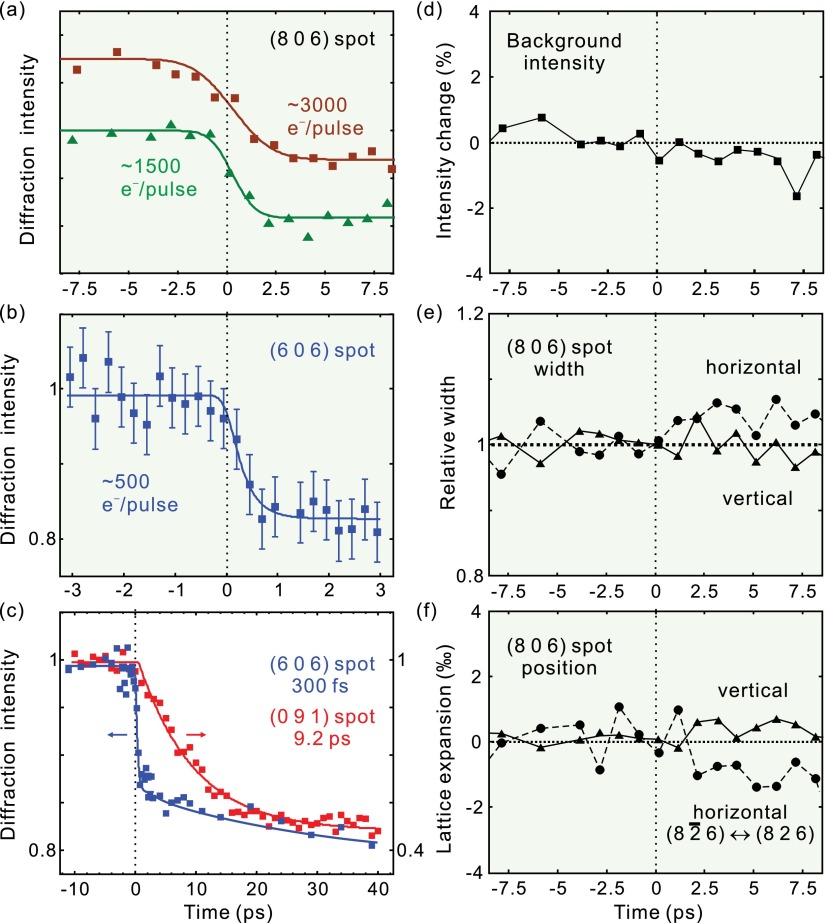
Ultrafast dynamics of the photoinduced structural phase transition of VO_2_. (a) Time-resolved intensity change of the (806)_m_ Bragg spot measured with relatively larger numbers of electrons per pulse. The ultrafast change is faster than the temporal resolution here. (b) Femtosecond intensity decrease of the (606)_m_ Bragg spot measured with ∼500 electrons per pulse. (c) Intensity changes of the (606)_m_ (blue) and (091)_m_ (red) spots with time. (d) Constant scattering intensity in the vicinity of the (806)_m_ spot as a function of time. (e) Vertical and horizontal width change of the (806)_m_ spot with time. (f) Negligible vertical expansion (derived from the position change of the (806)_m_ spot) and horizontal expansion (from the spacing between the (8 $\bar{2}$ 6)_m_ and (826)_m_ spots) as a function of time.

All intense-enough Bragg reflections were fitted numerically for their intensities, positions, and widths as a function of time. Figure 4 summarizes the results. Figure 4(a) depicts the time-dependent intensity of the (806) Bragg spot for a decreasing number of electrons per pulse, which demonstrates a clear improvement of the temporal resolution. At ∼500 electrons per pulse (Fig. 4(b)), the trace allows a fit of an exponential decrease convoluted with the independently determined electron-pulse duration (322 fs), revealing a time constant of ∼300 fs (Ref. 5) that was later confirmed again.^6^ Figure 4(c) shows in comparison the time-dependent intensity of the (091) spot with the (606) spot, which reveals a decay that lacks such fast dynamics, but changes intensity only within ∼10 ps.

The femtosecond behavior is evident in the following Bragg spots: (8*k*6) shown in Fig. 2(c) with *k* = 0, ±1, ±2, ±3 or ±4; also in (10 2 4), (915), (806), (7$\bar{1}$7), (6$\bar{2}$8), (10 1 6), (907), (8$\bar{1}$8), (923), (814), and (5$\bar{2}$7) in Fig. 2(d), and in (606), (714), and (4$\bar{1}$7) in Fig. 2(e). In contrast, the slower picosecond behavior is not affected by the electron pulse width and is observed for the (091), (084), and (08$\bar{2}$) Bragg spots from the single crystal and also for the (040) spot from the thin-film sample (see below).

Bragg spot intensity changes at constant Bragg condition originate from atomic motions within the unit cell, via a time-dependent modification of the atomic scattering interference, i.e., the structure factor. The inner product between the atomic positions and Miller indices in the structure factor makes it evident that an atomic movement along a certain direction can only affect those Bragg spots with nonzero components in the corresponding Miller indices. Thus, it is concluded from the experimental results that the initial femtosecond motion is mostly along the *a*_m_ axis, which corresponds to the direction of the V–V bonds in the *M*_1_ structure.^5^ The V atoms do not move directly towards their final positions, which would be motions along mostly the *c*_m_ axis (see Fig. 1(d)). In contrast, the ps dynamics deduced from the (091) spot (red trace in Fig. 4(c)) provide the structural changes predominantly along the *b*_m_ and *c*_m_ axes. Hence, the photoinduced phase transformation of VO_2_ involves not only the initial and final states but also transitional structures appearing on the fs and ps time scales, one of which is with dilated or broken V–V pairs in the monoclinic unit cell.^5^ Essentially, VO_2_'s laser-triggered phase transition therefore proceeds through a non-direct, sequential pathway involving multidimensional reaction coordinates and multiple time scales, hence with some similarity to a chemical reaction.

The observed femtosecond V–V bond dilation can be described from such a chemical perspective by considering that the initiating excitation at 1.55 eV is primarily into the d_||_ band that contributes to the dimer bonding.^35,36^ Directly after femtosecond absorption, but after the electronic correlation has stabilized on a few-fs time scale,^9,11,19^ this band has an antibonding character and causes a repulsive force on the atoms, probably in conjunction with bond softening.^5^ Consequently, the V–V dimers separate/delocalize along the bond direction, within an apparent time of ∼300 fs as observed. Subsequently, over several ps, all atoms in the unit cell adjust themselves towards the intracell configuration of the tetragonal phase, but still constrained macroscopically to the lattice orientation and cell dimensions of the monoclinic phase. Only on much slower time scales (∼100 ps), the thermodynamically stable, final structure is reached (see below). We note that a previous study with time-resolved X-ray diffraction^37^ showed results that are consistent with this picture, by revealing a 12-ps time scale and only a very weak fs contribution in the (011) spot^38^ in a direction perpendicular to the dimers. Subsequent time-resolved X-ray diffraction studies also confirmed this picture.^39,40^

Additional support measurements and analyses are shown in Figs. 4(d)–4(f). First, we studied the time dependence of the inelastic scattering background, which could originate from the Debye-Waller effect or other random delocalizations in the crystal structure. The time-dependent electron background intensity, evaluated in the vicinity of the (806) spot, exhibits essentially no dynamics and maintains at a constant level (Fig. 4(d)). This indicates that the ∼20% intensity decrease in Figs. 4(a)–4(c) is indeed from the directed atomic motion and not from losses of the structural correlation. Second, we evaluated the time-dependent widths of the Bragg spots, which could become broader due to domain formation and loss of crystalline order. Figure 4(e) shows the fitted spot widths in horizontal and vertical direction of the (806) reflection, revealing no significant dynamics. Thus, it is concluded that domain formation is insignificant, at least on the length scale of the electron beam's coherence widths,^41^ which is, according to the Bragg spot widths in horizontal direction, about 5 nm at the sample. Third, we monitored the Bragg spot centers, which would change position if there were a time-dependent expansion or contraction. Figure 4(f) shows that such effects stays below 0.1% at ps times, indicating that all laser-induced structural motion happens almost exclusively as atomic rearrangements within the unit cell of the monoclinic phase. For comparison, a clear Bragg spot movement^42^ as large as 2.5% and width broadening^22^ of more than 50% have been observed in other laser-excited materials. The observed significant Bragg spot intensity decreases together with insignificant lattice changes show the intracell nature of the fs and few-ps dynamics in VO_2_ and the existence of metastable transitional structures, i.e., an indirect reaction pathway via laser-induced, thermodynamically “hidden” states. Based on optical reflectivity and THz transmission experiments, VO_2_ can become metallic within ∼80 fs,^17,18^ before involvement of the phonons at 6 THz.^7,9^ In contrast, the measured primary step in the structural dynamics is ∼300 fs; whether a faster, weaker atomic motion component remains hidden or whether coherent phonons involve in the excited state^7,9^ will be subject to upcoming diffraction studies with compressed electron pulses and few-fs time resolution.^43–45^

**FIG. 5.**
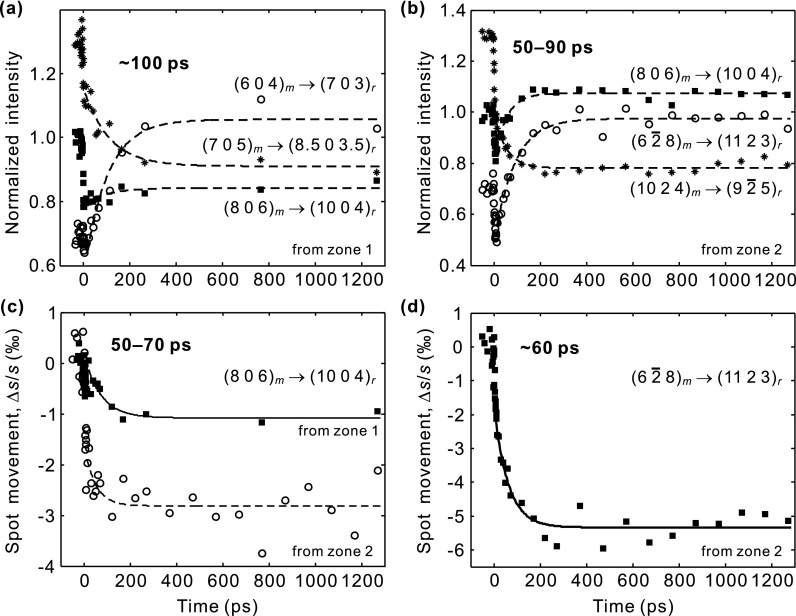
Long-time change in (a) and (b) the diffraction intensities and (c) and (d) the positions, with constant vertical offsets for clarity. The temporal profiles are fitted to a single exponential decay function, and the extracted time constant is given in each panel. After the phase transformation, the diffraction intensities and positions remain for >1 ns.

Figure 5 reports the structural dynamics of VO_2_ on longer time scales. If sufficient energy is deposited, the VO_2_ structure eventually evolves into the thermodynamically stable, tetragonal form. This includes completion of the intracell atomic motion and also the adjustments of the lattice vectors and angles via shear dynamics.^5^ The common feature is the evolution of the diffraction intensity and position change with a time constant of 50–100 ps. This structure is then stable for at least 1 ns, the longest delay measured. For different spots, either intensity increases or decreases are observed (Figs. 5(a) and 5(b)), depending on the diffraction indices and also the overlap between the reciprocal lattice points and the Ewald sphere after the structural transformation; see below. For the spot positions, plotted in Figs. 5(c) and 5(d), we observe ∼0.1% and ∼0.5% decrease in the reciprocal vectors of the (806)_m_ and (6$\bar{2}$8)_m_ spots, respectively. This fits reasonably to the lattice-vector length changes expected thermodynamically for these two spots, −0.23% to 0.33% and −0.19% to 0.44%, depending on the unit-cell parameters assumed;^14,46^ note that the experiment is also susceptible to the ∼0.5% tilts of the main axis directions^16^ via the rocking curve. The reasonable agreement between the measured lattice vector expansion/contraction with a common rate of 50–100 ps shows that the thermodynamically stable, tetragonal structure of VO_2_ is reached within ∼100 ps after laser excitation.

The origin of this time scale is macroscopic lattice expansion/contraction and shear motion of the laser-excited volume. The two thermodynamically stable phases below and above the transition temperature have slightly different unit cell volumes, somewhat different aspect ratios, and also the crystallographic angles differ by ∼0.5°.^16^ Overall, a shear motion is required to transform a macroscopic volume between the two phases. The optical penetration depth and hence the photoexcited thickness is ∼100 nm. Although only the topmost rough layer is effectively probed by diffraction, the inertia and dynamics of the whole excited volume determines the shear speed on the top. This nanoscale motion is difficult to model theoretically, but the estimate of a speed of sound of ∼4000 m/s in VO_2_ and a 100-nm excitation depth leads to a time scale of tens of ps, matching the observation. The relevance of this shear motion in the phase transition is further supported by the concurrent intensity and position changes of almost all Bragg spots on this time scale. Crystallographic main-axis adjustments change the overlap of the reciprocal space with the Ewald sphere (rocking curve), which produces spot displacements and intensity changes at the same time and in time-dependent proportion.

As discussed above, the experiment reveals that the VO_2_ crystal comes entirely back to the low-temperature phase within at least 1 ms in a completely reversible way. This back reaction speed is governed by how fast the laser-excited metallic phase at the top layer can lose its energy into the remaining bulk material, which is still mostly insulating. The thermal diffusivity coefficient of VO_2_ at *T* = 300 K is $\kappa =K/\rho C_{l}$ ≈ 0.021 cm^2^/s, where ρ = 4.67 g/cm^3^ is the density, $C_{l}$ = 0.67 J/(g K) is the specific heat, and *K* = 0.066 W/(cm K) is the thermal conductivity.^2^ Considering heat conduction in one dimension, i.e., from a surface layer into bulk,^47^ we obtain a time scale of $t\approx D^{2}/(4\kappa )\approx 1.2$ ns for a *D* = 100 nm thick layer. The actual process can take somewhat longer, for example, if there is limited thermal conductivity at the metal-insulator domain boundaries, but will be completed in ≪1 *μ*s and certainly ≪1 ms, the experimental upper limit. This fast relaxation is a consequence of the bulk crystal's macroscopic shape, and also marks a practical advantage of probing in grazing-incidence diffraction geometry vs. studying free-standing membranes in transmission.

### B. Crystalline VO_2_ thin-films

While single crystals are ideal to study the basic reaction path, deposited thin-films on substrates have more flexible technological applications. Here, we therefore report an ultrafast electron crystallography study on single-crystalline VO_2_ films with nanometer thickness. To avoid impurities, inhomogeneities, domains, and strain, which can substantially alter the transition temperature and reaction path, largely single-crystalline VO_2_ thin-films on various sapphire wafers were prepared via pulsed laser deposition (PLD).^48–51^ A KrF excimer laser (COMPex Pro 102, Coherent, Inc.) with ∼300 mJ per 20-ns pulse ablated a rotating target of pure vanadium metal (99.9%, ESPI Metals) at a repetition rate of 20 Hz. Single-crystalline sapphire substrate wafers (EPI-polished, MTI Inc.) were held at a temperature of 500 °C during the film growth at a constant flow of a 10% oxygen and 90% argon mixture at 28 mTorr. The typical target-substrate distance was 9 cm. The thickness of the VO_2_ films after 10^5^ laser pulses was ∼140 nm, measured by a contact profilometer. To enhance homogeneity, domain size, and connectivity, the deposited films were annealed for 42 h in a pure oxygen atmosphere at 10 mTorr. The phase transition was observed by infrared transmission at 2‐*μ*m wavelength (0.62 eV) as a function of temperature. The closing of the band gap at high temperature^2^ leads to a 30%–45% decrease in transmittance, with a hysteresis width of ∼6 K.

Among the samples prepared on the Al_2_O_3_ wafers with different surface planes, we found that VO_2_/Al_2_O_3_(10$\bar{1}$0) was able to produce the nicest electron diffraction patterns at an intermediate level of roughness, whereas VO_2_ on Al_2_O_3_(0001) and VO_2_ on Al_2_O_3_(1$\bar{1}$02) often produced very transmission-like diffraction patterns, indicating isolated islands. Nevertheless, all VO_2_ films were highly oriented over mm distances. Here, we report the results of VO_2_/Al_2_O_3_(10$\bar{1}$0).

**FIG. 6.**
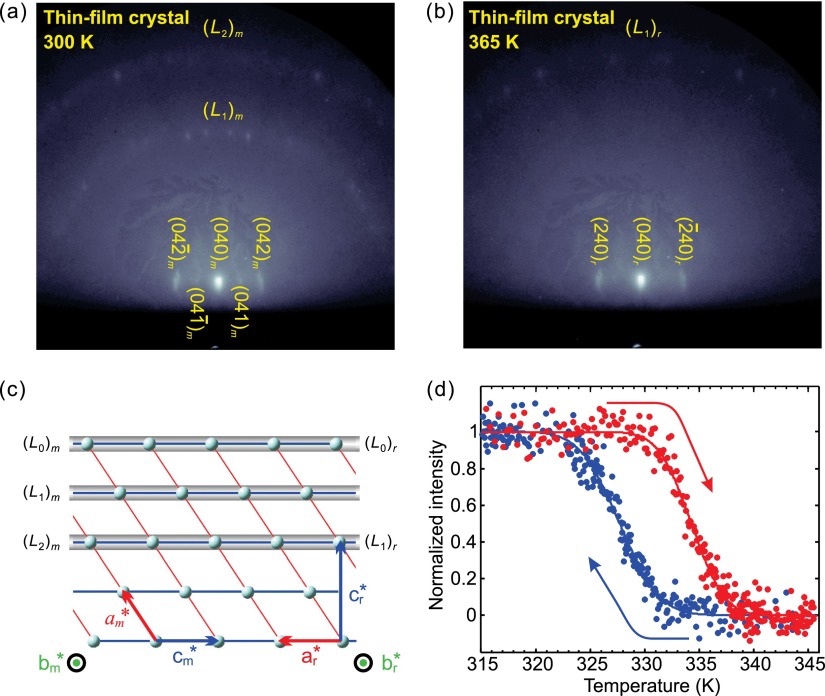
Diffraction of single-crystalline thin-films of VO_2_. (a) Reflection diffraction pattern of a VO_2_ thin-film on Al_2_O_3_(10$\bar{1}$0), in the low-temperature *M*_1_ phase. Bragg spots from the zeroth-order Laue zone, (*L*_0_)_m_, are indexed and the first- and second-order Laue zones [(*L*_1_)_m_ and (*L*_2_)_m_, respectively] are indicated. (b) Diffraction pattern of the high-temperature phase. Note the missing Laue zone. (c) Relationship between the reciprocal lattices for the two phases. In the high-temperature phase, (*L*_1_)_m_ is forbidden and therefore disappears. (d) Diffraction intensity of the four (*L*_1_)_m_ spots in the center as a function of temperature during the heating (red dots) and cooling (blue dots) cycles.

Figures 6(a) and 6(b) show the electron diffraction patterns at an incidence angle of 1.75° and for two temperatures, 300 and 365 K, respectively. The electrons probed along the *c* axis of the tetragonal phase (see Fig. 6(c)). From the Bragg spots, including those noticeable at higher-order Laue zones, we confirm that the (010) plane of VO_2_ is parallel to the wafer plane (10 $\bar{1}$ 0) and that we indeed have a highly crystalline specimen. The structural transformation can be clearly seen by comparing low-temperature and high-temperature diffractions (Figs. 6(a) and 6(b)), as evidenced in the disappearance of the first-order Laue zone diffractions that originate from reciprocal rods with the index of *h* = 1 upon specimen heating above the transition temperature. The temperature dependence of the total intensity of the four spots near the center of the first Laue circle (Fig. 6(a)) reveals a phase transition at ∼334 K and a hysteresis width of ∼6 K (Fig. 6(d)). These values are satisfactorily consistent with those retrieved from the infrared transmission data.

**FIG. 7.**
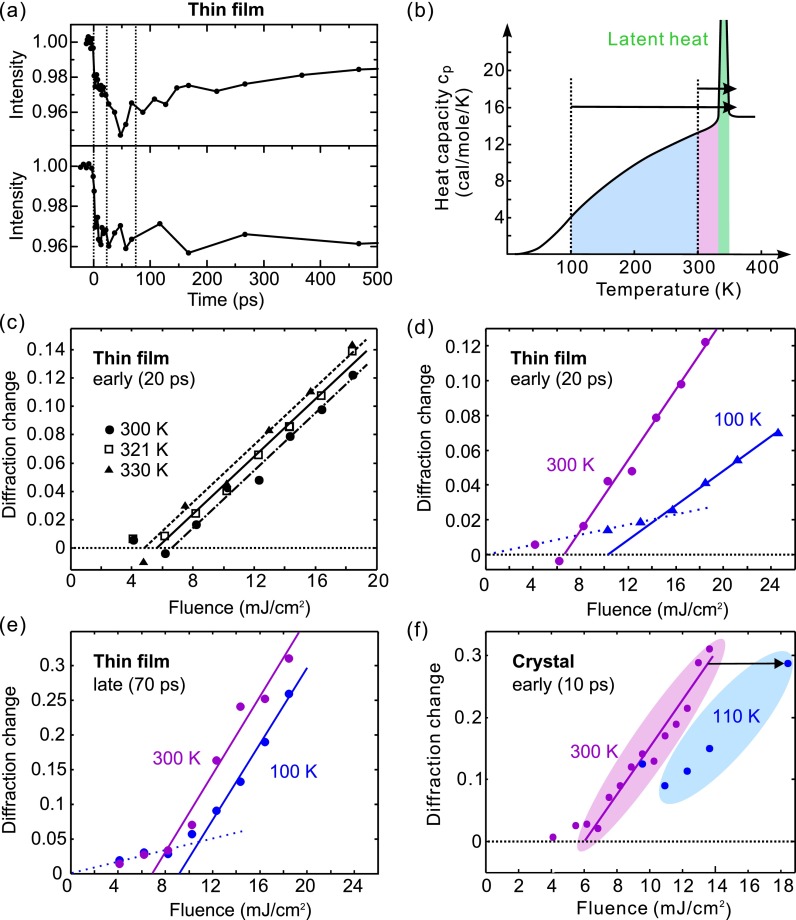
Excitation fluences and the photoinduced phase transition of VO_2_. (a) Time-resolved intensity change of the (040)_m_ Bragg spot in thin-film VO_2_/Al_2_O_3_(10$\bar{1}$0), at 11 mJ/cm^2^ at room temperature (upper panel) and at 19 mJ/cm^2^ at 100 K (lower panel). (b) Heat capacity of VO_2_ as a function of temperature (solid curves, adapted from Ref. 2). The colored areas indicate the energies required thermodynamically at different starting temperatures (dashed lines) for the phase transition, including the latent heat (green). (c) and (d) Fluence dependence of the early-time diffraction intensity decrease (measured at *t* = 20 ps) for selected ambient temperatures. (e) Fluence dependence of the longer-time intensity decrease (measured at *t* = 70 ps) for the thin-film specimen. (f) Fluence dependence of the early-time intensity decrease for VO_2_ bulk. The arrow indicated the required fluence increase to reach a similar level of diffraction change as at room temperature.

Figure 7(a) shows the time-resolved dynamics of the (040) Bragg spot for the thin-film sample at an excitation fluence of ∼10 mJ/cm^2^ and at two ambient temperatures, 300 K (upper panel) and 100 K (lower panel). Electron pulses of ∼3000 electrons were applied, favoring sensitivity over temporal resolution. Evident for both initial temperatures, as before for the single crystal, is a prompt intensity decrease (few-ps, resolution-limited), followed by a slower ∼50–100-ps dynamics and a saturation afterwards. The reaction is again fully reversible within the 1-kHz repetition rate of the pump-probe experiment. Overall, the thin-film dynamics is very similar to the single-crystal results.

### C. Temperature-dependent excitation fluence thresholds

Triggering the phase transformation of VO_2_ with laser excitation should eventually lead to the final, tetragonal phase via the established sequence of structural events, if the total deposited energy is enough to overcome the latent heat and reach a thermodynamically stable phase. In our initial report,^5^ we considered the question of how this minimum deposited energy is related to the observed fluence threshold for laser-induced, early-time fs and ps dynamics. Such a comparison was prompted by the proximity of the total energy deposited at the apparent threshold of 6 ± 1 mJ/cm^2^ (about 1800 ± 300 cal/mol at the surface) and the sum of heat required thermodynamically for reaching the transition and overcoming the latent heat from room temperature, in total 1570 cal/mol. We note that the temperature-dependent heat capacity (*c_p_*) of VO_2_ fits very well to a Debye temperature of 750 K;^2^ the latent heat (the green area in Fig. 7(b)) is 1020 cal/mol or 235 J/cm^3^.^2,10^ The applicability of the Debye model with a single temperature indicates that the phonon modes contributing to the specific heat are not significantly different below and above the transition temperature.

Temperature- and time-dependent threshold measurements can clarify this picture. Far-infrared and multi-THz pump-probe spectroscopy, techniques sensitive to the insulator-metal transition but not directly visualizing the atomic motion, had revealed for decreasing sample temperature a higher fluence threshold,^7–10^ which seems to indicate a picture of energetics that coincides with thermodynamics.^9^ Whether the structural aspect of the phase transition exhibits a similar behavior is now investigated.

We first report the thin-film results (see Figs. 7(c)–7(e)); the single-crystal case is discussed below (Fig. 7(f)). Basically, the structural dynamics of crystalline VO_2_ thin-films follows the basic reaction path established above (Fig. 1(d)), including a few-ps prompt response and slower sound-wave dynamics towards a constant at ∼1 ns. Intriguingly, the intensity amplitudes at different temperatures show notable differences. Thus, to elucidate the influence of the ambient temperature on the structural phase transformation, diffraction intensity changes were monitored as a function of temperature at two characteristic times: at 20 ps, when the intracell atomic movements of the V–V bond dilation and the oxygen octahedron adjustment have mostly completed, and at 70 ps, when the sound-wave shear motion and lattice reorientation are largely complete. Although the (040) Bragg spot investigated here does not have a direct projection of the *a*_m_-axis along which the femtosecond V–V dilation primarily occurs, it well serves as an indicator of how much of the transitional state structure is present after 20–70 ps as a function of the laser excitation fluence and specimen temperature. Figure 7(c) shows the amount of Bragg diffraction intensity decrease for different excitation fluences and temperatures, measured at the VO_2_/Al_2_O_3_(10$\bar{1}$0) thin-film sample at a 20-ps delay. Going with the specimen temperature from 300 K to 330 K, a trend of the threshold towards lower values is revealed, which is consistent with the expectation for less energy required for the phase transition at a higher temperature. Figure 7(d) shows the results of a more extreme change of temperature, achieved by liquid-nitrogen cooling of the thin-film sample within the electron diffraction chamber. First, the room-temperature data (dots) closely resembles the bulk-crystal results,^5^ confirming again the general nature of the reaction path in different specimen morphologies. Second, the fluence threshold at 100 K (triangles) is strongly displaced towards higher values, maybe with a weak lower-threshold contribution (dotted). Figure 7(e) shows the fluence threshold evaluated later, at a 70-ps delay, where sound-wave shear motion and lattice reorientation is largely complete (compare Fig. 5). Also here, there is a clear shift of the room-temperature threshold (violet) towards higher levels with decreasing temperature (blue). Finally, we also managed to re-measure the bulk crystal's structural dynamics at cryogenic temperatures, which was difficult due to the macroscopic thermal stress and ultimately fragmentation after several millions of phase-transition and back-reaction cycles. Figure 7(f) shows the threshold results taken at 110 K (blue) and at 10-ps delay, again revealing, qualitatively, the expected shift with temperature (black arrow) as compared to the 300 K results (violet).

At room temperature and 800-nm excitation, there is a striking coincidence^5^ between the thermodynamical heat required to overcome the phase transition including the latent heat (see Fig. 7(b)) and the optically deposited total energy needed at the laser fluence threshold required to initiate the structural conversion of VO_2_. Both energies are very similar. On the one hand, the temperature-dependent threshold results reported here further support such a connection. On the other hand, at early times and at the fluence threshold, only an average of ∼0.05 photon per vanadium ion, or one photon for every 10 dimers, is sufficient to trigger the transformation. Below the threshold, the V–V bond is set on a coherent vibrational motion^7,9^ without promoting the structural symmetry change. These two observations, one in a photon-per-dimer picture and the other one from a quasi-equilibrium perspective, indicate a delicate interplay between the initial localization of a photon into a set of VO_2_ unit cells and the cooperative forces of the crystal potential promoting the irreversible reaction.

## V. CONCLUSION

The key results of this study are twofold. First, the photoinduced structural phase transition of VO_2_ has a stepwise transformation mechanism in almost any sample morphology, provided a minimum of single-crystalline nature and correct stoichiometry. Second, the lower the ambient temperature, the more fluence or photo-doping is required to overcome the cooperativity threshold for the structural transformation.

These results, made with a time-resolved electron diffraction technique that directly monitors the atomic motions and potentially also charges,^52^ will help strengthening our general understanding of photoinduced phase transitions in complex materials, where the time-dependent hierarchy of electronic and structural reaction steps is often the key to disentangle the correlations between charges, spins, lattice motions, and other atomic-scale degrees of freedom.
